# Metabolic reprogramming and renal fibrosis: what role might Chinese medicine play?

**DOI:** 10.1186/s13020-024-01004-x

**Published:** 2024-10-28

**Authors:** Weili Wang, Rong Dai, Meng Cheng, Yizhen Chen, Yilin Gao, Xin Hong, Wei Zhang, Yiping Wang, Lei Zhang

**Affiliations:** 1 First Clinical Medical College, Anhui University of Chinese Medicine, Hefei, China; 2 Department of Nephrology, The First Affiliated Hospital of Anhui University of Chinese Medicine, Meishan Road 117, Shushang District, Hefei, 230031 China

**Keywords:** Chinese medicine, Metabolic reprogramming, Renal fibrosis

## Abstract

Metabolic reprogramming is a pivotal biological process in which cellular metabolic patterns change to meet the energy demands of increased cell growth and proliferation. In this review, we explore metabolic reprogramming and its impact on fibrotic diseases, providing a detailed overview of the key processes involved in the metabolic reprogramming of renal fibrosis, including fatty acid decomposition and synthesis, glycolysis, and amino acid catabolism. In addition, we report that Chinese medicine ameliorates renal inflammation, oxidative stress, and apoptosis in chronic kidney disease by regulating metabolic processes, thereby inhibiting renal fibrosis. Furthermore, we reveal that multiple targets and signaling pathways contribute to the metabolic regulatory effects of Chinese medicine. In summary, this review aims to elucidate the mechanisms by which Chinese medicine inhibits renal fibrosis through the remodeling of renal cell metabolic processes, with the goal of discovering new therapeutic drugs for treating renal fibrosis.

**Supplementary Information:**

The online version contains supplementary material available at 10.1186/s13020-024-01004-x.

## Introduction

### Metabolic reprogramming

Metabolic reprogramming, which was first observed in tumor cells by Otto Warburg, is characterized by an increase in glucose metabolism, also known as glycolysis. This phenomenon, termed the Warburg effect, persists even in the presence of oxygen and is characterized by increased glucose uptake and lactate production [1]. Further investigations have revealed metabolic reprogramming to be a biological process during which the energy metabolic patterns of cells, such as those associated with glucose, lipids, and amino acids (AAs), are altered to support cell survival and growth [2]. The mechanism of metabolic reprogramming encompasses four main facets. The first is the enhanced Warburg effect of glycolysis, where tumor cells shift their primary glucose metabolism from oxidative phosphorylation (OXPHOS) to aerobic glycolysis to meet their energy demands [3, 4]. Second, the modulation of key enzyme expression or activity alters cellular metabolism to cater to growth and proliferation requirements [5, 6]. Third, nutrient availability and oxygen concentration exert significant effects [7, 8]. Nutrient effectiveness is a driving factor of metabolic reprogramming; when insufficient substrate is available for metabolism in the cellular microenvironment, the cell uses other energy metabolism pathways to meet metabolic demand [9, 10]. However, changes in oxygen concentration mainly affect OXPHOS and the expression of key metabolic enzymes, such as hypoxia-inducible factor-1α (HIF-1α) and phosphofructokinase [11, 12]. The reprogramming of AA and fatty acid metabolism is another aspect of metabolic reprogramming, and it may involve specific alterations in AA or fatty acid metabolism pathways. These mechanisms collectively enable cells to adapt to external environmental changes, thereby ensuring their survival and proliferation.

### Metabolic reprogramming and fibrosis

From the perspective of cell types, metabolic reprogramming can occur in both tumor and non-tumor cells, including immune cells, fibroblasts, and epithelial cells, and even in pathogens [2, 13–15]. Metabolic changes are increasingly recognized as critical pathogenic processes and hallmarks of fibrosis in various organs. Fibrosis involves the abnormal deposition of fibrous extracellular matrix (ECM), leading to an increase in fibrous tissue and a decrease in the number of parenchymal cells in organs and tissues such as the lungs, heart, liver, and kidneys [16]. This pathology is characterized by a progressive and continuous process of damage repair. Prolonged fibrosis can result in the structural destruction of organs, the functional deterioration of tissues, and even organ failure [17].

Cellular metabolic reprogramming plays a significant role in the pathological process of pulmonary fibrosis. Lipopolysaccharide promotes aerobic glycolysis in pulmonary fibroblasts by activating the PI3K-Akt-mTOR/6-phosphofructo-2-kinase/fructose-2,6-biphosphatase 3 (PFKFB3) signaling pathway, which promotes collagen synthesis in pulmonary fibroblasts and contributes to pulmonary fibrosis [18]. In contrast, the CAV1 scaffolding domain peptide or its 7-amino acid deletion fragment (CSP/CSP7) restores the expression of p53 and miR-34a, inhibiting HIF-1α and glycolysis and regulating abnormal glucose metabolism in fibrotic lung fibroblasts, consequently inhibiting pulmonary fibrosis [19]. Furthermore, glutaminolysis and glutamate-derived AA biosynthesis are essential components of metabolic reprogramming and collagen production in myofibroblasts differentiated from pulmonary fibroblasts [20]. TGF-β1 upregulates glutaminase expression by activating the Smad3 and p38 mitogen-activated protein kinase (p38-MAPK) signaling pathways, thereby stimulating pulmonary myofibroblast glutaminolysis and accelerating pulmonary fibrosis [21]. Fatty acid oxidation (FAO) also plays a significant role in the pathogenesis of pulmonary fibrosis. The mitochondrial calcium uniporter (MCU) increases the expression and activation of peroxisome proliferator-activated receptor γ coactivator 1α (PGC-1α) by activating the p38-MAPK signaling pathway while also enhancing FAO in lung macrophages, thereby promoting the progression of fibrotic repair [22].

Myofibroblast activation is an important factor in cardiac fibrosis. Methyltransferase-like 3 (METTL3) facilitates glycolysis and cardiac fibroblast proliferation by increasing methylation of the androgen receptor (AR) in an m6A-YTHDF2-dependent manner while also enhancing the expression of HIF-1α and the glycolytic enzyme HK3. In contrast, knockdown of METTL3 suppresses glycolysis and inhibits cardiac fibroblast proliferation and cardiac fibrosis [23]. This suggests that inhibiting glycolysis and blocking related activation molecules may help prevent the occurrence of cardiac fibrosis.

Liver fibrosis develops from chronic liver inflammation. Macrophages, the key regulators of liver inflammation, dominate the progression and regression of liver fibrosis [24, 25]. Follistatin-like protein 1 (FSTL1) is thought to be a proinflammatory gene involved in macrophage polarization that accelerates the progression of liver fibrosis by promoting pyruvate kinase M2 (PKM2) phosphorylation and inhibiting PKM2 ubiquitination in macrophages, thereby increasing M1 polarization, glycolysis, and inflammatory responses [26]. In contrast, inhibiting glycolysis and promoting OXPHOS attenuates fibrosis symptoms in liver fibrosis. Glutaminase expression is downregulated by inhibiting the hedgehog-yes-associated protein 1 signaling pathway, which in turn inhibits glutamine catabolism in myofibroblast stellate cells, reduces myofibroblast activity in hepatic stellate cells, and blocks the accumulation of myofibroblasts and the progression of fibrosis [27].

**Fig. 1:**
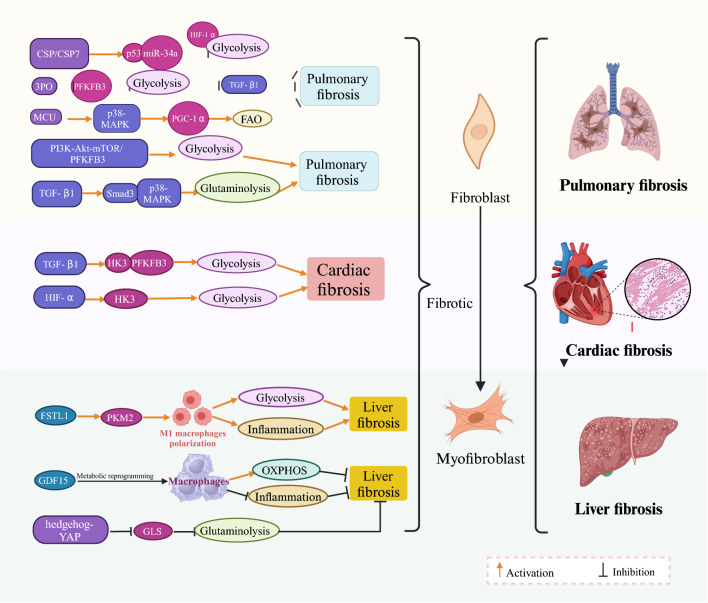
Mechanism of metabolic reprogramming in fibrotic diseases

In summary, cellular metabolic reprogramming plays a crucial role in the pathological progression of fibrosis. Glycolysis, FAO, and glutamine metabolism are particularly closely linked to the activation and proliferation of fibroblasts, a phenomenon also observed in renal fibrosis. Next, we explore the relationship between metabolic reprogramming and renal fibrosis in detail. The specific regulatory mechanism of metabolic reprogramming in fibrotic diseases is illustrated in Fig. 1.

### Metabolic reprogramming and renal fibrosis

CKD has become a crucial public health problem worldwide, resulting in a considerable socioeconomic burden. Hypertension and diabetes mellitus are the most common pathogenic factors for CKD [28]. However, renal fibrosis is the most common pathway in many types of CKD, and all CKD patients experience pathological changes associated with renal fibrosis [29], which manifests microscopically as glomerulosclerosis, tubular injury, interstitial fibrosis, and capillary thinning. Renal fibrosis is characterized by loss of the capillary network, the accumulation of fibrillar collagen, an interstitial inflammatory response, and interstitial fibroblast activation [30]. Recent studies have shown that metabolic reprogramming occurs in renal cells after kidney injury [31]. Renal fibroblasts and tubular epithelial cells (TECs) are the main cell types that undergo metabolic reprogramming during renal fibrosis [32]. Reprogramming of lipid, glucose, and AA metabolism occurs in CKD, affecting the function and survival status of the aforementioned cell types. Regulating the metabolic reprogramming of renal cells can improve renal function, inhibit renal fibrosis, and slow the progression of renal disease.

**Fig. 2:**
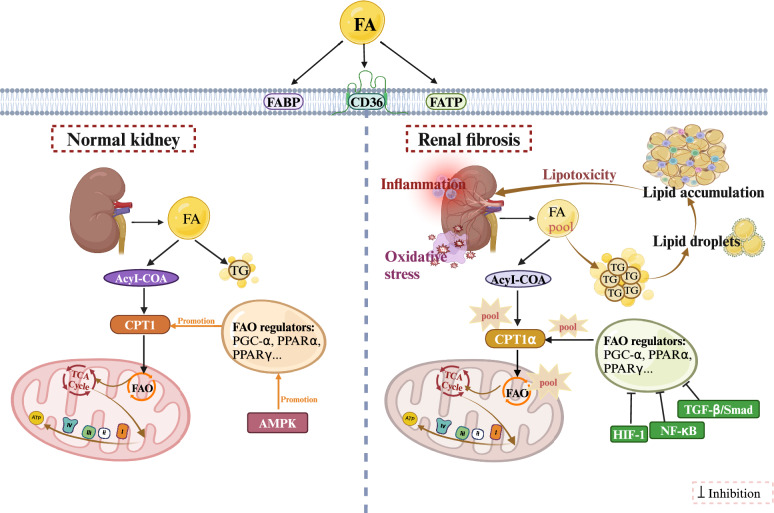
Mechanism of lipid metabolism in the normal kidney and in renal fibrosis

Changes in lipid metabolism are closely related to renal fibrosis. FAO is the optimal energy supply mode for renal cellular activity, and most fatty acids are generated via FAO, producing adenosine triphosphate (ATP) to supply energy to TECs. In contrast, uncatabolized fatty acids are typically stored as triglycerides. When FAO is inhibited, fatty acid decomposition decreases and the triglyceride content increases. When the lipid metabolism ability of nonadipose cells is limited, the abnormal accumulation of lipids will cause there to be an excessive number of lipid droplets in cells [33]. This response leads to cellular lipotoxicity and promotes the development of renal fibrosis [34, 35]. Therefore, we can regulate lipid metabolism by activating FAO activity and attenuating intrarenal lipid accumulation to inhibit renal fibrosis progression. Figure 2 shows the mechanism of lipid metabolism in the normal kidney and in renal fibrosis.

**Fig. 3:**
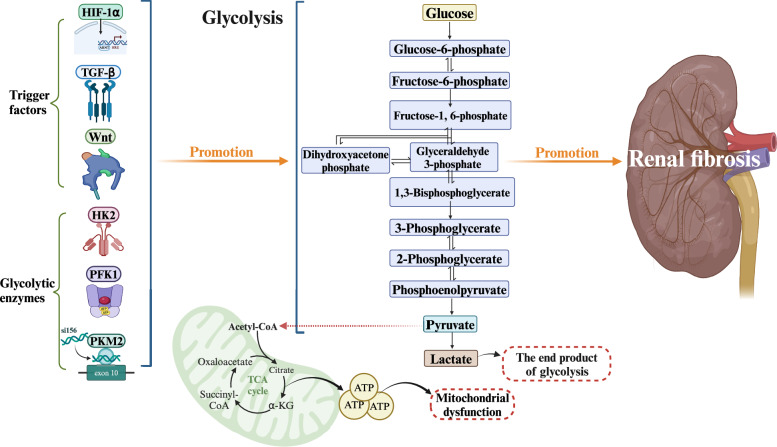
Mechanism of glycolysis in renal fibrosis

Furthermore, the pattern of glucose metabolism changes in renal cells. Studies have shown that aerobic glycolysis occurs in renal injury and tumor cells. When the metabolic pattern of renal interstitial fibroblasts is converted from OXPHOS to aerobic glycolysis, fibrosis and proliferation indices are increased, further exacerbating CKD [36]. Moreover, during renal fibrosis, several signaling pathways and glycolysis-related enzymes, such as the TGF-β, HIF-1α, and PI3K/Akt pathways [37, 38], hexokinase 2 (HK2), phosphofructokinase-1 (PFK1), and PKM2, are activated [39–41]. The activation of these pathways and enzymes may enhance glycolysis and further aggravate renal fibrosis. Furthermore, the release of HIF-α can stimulate TECs and glomerular endothelial cells, thereby enhancing glycolysis [42]. As a result, reducing glucose uptake and lactic acid production by inhibiting glycolysis in renal tissues and cells is crucial for attenuating renal fibrosis. Figure 3 shows the mechanism of glycolysis in renal fibrosis.

**Fig. 4:**
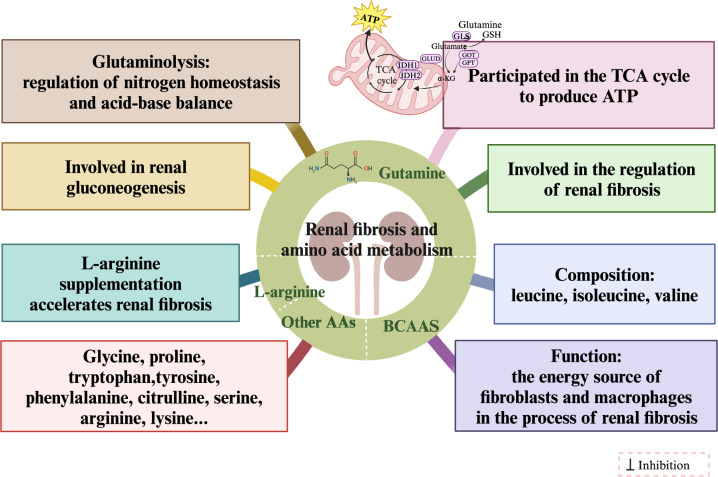
Mechanism of amino acid metabolism in renal fibrosis

Similarly, AA metabolism is essential in renal fibrosis. As the most abundant AA in the body and a substrate for protein synthesis, glutamine is involved in several metabolic pathways and plays a particularly crucial role in renal fibrosis. For example, glutamine can prevent apoptosis in human proximal TECs by inducing heme oxygenase-1 via the p38-MAPK pathway [43]. Glutamine can also be converted into α-ketoglutarate by catabolism in the tricarboxylic acid (TCA) cycle, producing energy in the form of ATP [44]. Additionally, silencing glutamine synthetase (GLS) can inhibit TGF-β1-mediated expression of fibronectin (FN) and alpha-smooth muscle actin (α-SMA), thereby decreasing glutamine catabolism and inhibiting renal fibroblast activation [45]. Branched-chain amino acids (BCAAs) such as leucine, isoleucine, and valine are important energy sources for fibroblasts and macrophages in renal fibrosis. BCAAs can affect renal fibrosis by targeting fibrotic signaling pathways to influence cellular autophagy, the inflammatory response, cell proliferation, and other processes [46]. Changes in the metabolism of AAs, such as glycine, tryptophan, tyrosine, phenylalanine, and proline, are also closely related to renal fibrosis [47]. Moreover, supplementation with certain specific AAs, such as L-arginine, may accelerate renal fibrosis and the loss of renal function [48]. Therefore, regulating AA metabolism, especially glutamine metabolism, may be another effective strategy for treating renal fibrosis. Figure 4 shows the mechanism of AA metabolism in renal fibrosis.

In addition, several signaling pathways play significant roles in metabolic reprogramming in CKD. Peroxisome proliferator-activated receptor gamma (PPARγ) signaling is involved in the expression of many genes, including renal genes. PPARγ also regulates glucose metabolism, insulin sensitivity, lipid metabolism, fibrosis, inflammation, and oxidative stress [49, 50]. Jagged1 and Notch2 can directly bind to mitochondrial transcription factor A (Tfam) to reprogram TEC metabolism and induce TEC dedifferentiation, proliferation, and fibrosis [51]. In contrast, some microRNAs, such as miR-9-5p, can inhibit chronic kidney injury and renal fibrosis by regulating TECs and mitochondrial metabolic reprogramming [52]. The long-chain noncoding RNA FABP5P3/miR-22 axis can ameliorate TGFβ1-mediated dysregulation of FAO and fibrotic changes in proximal TECs [53].

### Chinese medicine regulates metabolic reprogramming to inhibit fibrosis

Recent studies have demonstrated that some pharmacologically active ingredients derived from Chinese medicine (CM) can inhibit fibrosis by modulating cellular metabolic pathways. Periplocymarin is an effective component of *Periplocae cortex* (Xiang jia pi) that improves AA and arachidonic acid metabolism by modulating the expression of endothelial-type nitric oxide synthase (eNOS) and cyclooxygenase-2 in cardiomyocytes. This results in the release of profibrotic metabolites being reduced, thereby inhibiting cardiac fibroblast transformation [54]. Tuberostemonine, the ctive ingredient of *Stemona tuberosa Lour* (Bai bu), downregulates hydroxyproline levels in lung tissue and significantly inhibits the excessive proliferation of lung fibroblasts. Furthermore, tuberostemonine enhances solute carrier family 7 member 11 (SLC7A11)/glutamate transporter expression, inhibits ferroptosis, and reduces inflammation and collagen deposition in the lung, thereby reducing pulmonary fibrosis [55]. Atractylenolide III, a natural compound isolated from the plant *Atractylodes macrocephala* Koidz. (Bai zhu), delays liver fibrosis progression by inhibiting the release of hepatic fibrosis-associated genes and reducing periportal collagen deposition by inhibiting the PI3K/Akt pathway and glutamine metabolism [56]. Ginsenoside Rb1, a dammarane-type triterpene saponin compound mainly distributed in *Panax ginseng* (Ren shen), has been demonstrated to ameliorate the aberrant expression of cardiac energy metabolism-related enzymes and promote cardiac FAO by inhibiting the Fas-associated death domain and activating the peroxisome proliferator-activated receptor alpha (PPARα) signaling pathway, which ultimately attenuates cardiac hypertrophy and cardiac fibrosis [57]. In liver fibrosis, ginsenoside reprograms intracellular glycolysis to reduce the migration and maturation of dendritic cells. Ginsenoside also promotes PD-L1 expression, reduces interleukin-12 (IL-12) secretion, and blocks CD8 + T cell and hepatic stellate cell activation through the PI3K-Akt-FoxO1 pathway in liver fibrosis, thereby alleviating liver injury and fibrogenesis [58].

## Role of Chinese medicine in the metabolic reprogramming of renal fibrosis

**Fig. 5:**
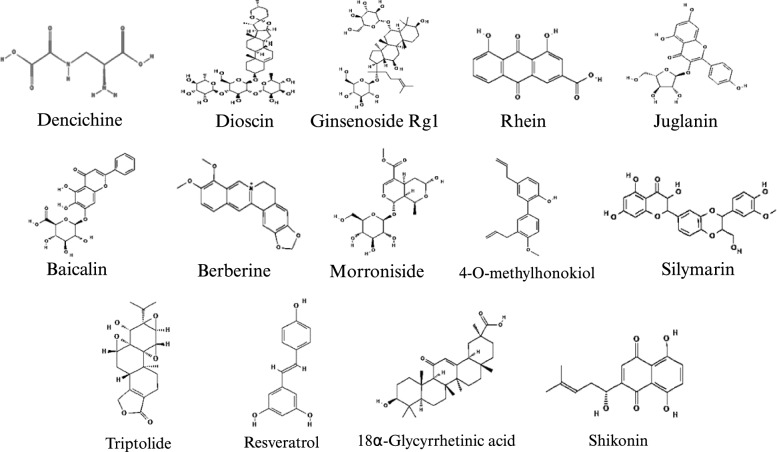
The chemical structures of the active ingredients used in CM that inhibit renal fibrosis through metabolic reprogramming

CM has been widely used as a complementary approach to CKD treatment in East Asia [59, 60]. At the macro level, CM can regulate the metabolism of renal cells by targeting multiple metabolic pathways and various targets. For example, metabolomics analyses have shown [61] that the surface layer of *Poria cocos* Wolf (Fu ling pi/FLP) reverses renal fibrosis in CKD by modulating fatty acid, phospholipid, AA, and adenine metabolism [62, 63]. N-butanol extract (BUT), an active ingredient of *Amygdalus Mongolia* (Yu li ren), improves renal tubulointerstitial injury and fibrosis by impacting arginine, proline, histidine, and nicotinamide metabolism [64]. *Rehmannia glutinosa* leaves (Di huang ye/DHY) primarily affect four metabolic pathways—ether lipid metabolism, sphingolipid metabolism, glyoxylic acid and dicarboxylic acid metabolism, and arachidonic acid metabolism—thus improving abnormalities in glucose and lipid metabolism and inflammatory responses [65]. This paper provides a detailed review of the effects and mechanisms of CM and its active components on fatty acid metabolism, lipid deposition, glycolysis, and amino acid catabolism during the process of renal fibrosis in CKD. The aim of this work is to enhance the understanding of the role of CM in renal fibrosis. Figure 5 shows the chemical structures of the active ingredients used in CM that inhibit renal fibrosis through metabolic reprogramming.

### Regulation of lipid metabolism

#### Lipid metabolism and renal fibrosis

Lipids are essential for the progression of renal fibrosis in CKD and play an important physiological role as a component of the cell membrane. Under normal circumstances, the contents of fatty acids, cholesterol, triglycerides, and phospholipids in cells are highly regulated, as these lipids are crucial for ensuring the structural integrity of cells and for signal transduction. However, an imbalance between fatty acid intake and utilization may result in lipid accumulation and kidney injury. When inflammation and oxidative stress persist, lipid accumulation worsens and foam cells develop; these cells, in turn, release cytokines, perpetuate lipid accumulation, and exacerbate lipid-mediated glomerular and interstitial injury [66]. The maladjustment of various lipids, such as fatty acids, cholesterol, triglycerides, and phospholipids, in podocytes, endothelial cells, and renal tubular cells may accelerate renal fibrosis in CKD [67]. Recent studies have shown that CM and its active ingredients can target the regulation of lipid metabolism, and these ingredients are promising inhibitors for renal fibrosis treatment. Tables 1 and 2 summarize the detailed regulatory mechanisms.

**Table 1:** Regulation of lipid metabolism by active ingredients used in Chinese medicine

Active ingredients	Herb source	Cell types	Mechanism	Effects	References
Dencichine	*Panax notoginseng* (San qi)	HBZY-1	Inhibition of the TGF-β/Smad2/3 pathway to attenuate inflammation	TGF-β1↓, Smad2↓, Smad3↓, Smad7↑, Col-I↓, Col-IV↓, FN↓, LN↓, MMP-9↑, α-SMA↓, Vimentin↓, E-Cad↑	[68]
Dioscin	*Dioscoreae Rhizoma* (Shan yao)	TECs	Regulation of the TGF-β/Smad pathway and activation of the Sirt3 pathway to attenuate inflammation and oxidative stress	TGF-β1↓, Smad3↓, Smad7↑, SREBP-1c↓, SCD-1↓, FASN↓, ACC↓, CPT1↑	[69]
Tartary buckwheat flavonoids	*Fagopyrum tataricum* (L.) Gaertn. (Ku qiao)	CMK-1	Inhibition of the TGF-β1/Smad, MAPK, and SREBP-1/FASN pathways and activation of the AMPK/ACC pathway to attenuate inflammation	TNF-α↓, IL-6↓, IL-1β↓, α-SMA↓, Vimentin↓, FN↓, E-cadherin↑, TC↓, TG↓, LDL↓, SREBP-1↓, HDL↑	[70]
Total *Rhizoma Coptidis* alkaloids	*Rhizoma Coptidis* (Huang lian)	TECs	Inhibition of the AGEs-RAGE-TGFβ/Smad2 and PI3K-Akt pathways to attenuate oxidative stress	Col1a1↓, Col1a2↓, Fn1↓, RAGE↓, TGF-β↓, p-Smad2↓, Smad2↓, TC↓, TG↓, HDL↑	[71]
Ginsenoside Rg1	*Panax ginseng* C. A. Mey. (Ren shen)	GMCs	Inhibition of the CD36/TRPC6/NFAT2 and TGF-β/Smad pathways to attenuate inflammation	CD36↓, PLC↓, TRPC6↓, CN↓, NFAT2↓, TGF-β↓, p-Smad2/3↓, Col- IV↓, FN↓	[72]
Rhein	*Rheum officinale* Baill. (Da huang)	TECs	Inhibition of the Sirt1/STAT3/Twist1 pathway to attenuate inflammation	CPT1↑, α-SMA↓, Col1a↓, TGF-β↓, Acta2↓, Vimentin↓, Twist1↓, Sirt1↓, STAT3↓, FN↓, E-cadherin↑	[73]
Juglanin	*Polygonum aviculare* L. (He shou wu)	HK2	Inhibition of the NF-κB and HDAC3 pathways to attenuate inflammation	TGF-β1↓, α-SMA↓, CTGF↓, FN1↓, Col1a1↓, Col1a2↓, SREBF1↓, FASN↓, SCD-1↓, ACCα↓, PPARγ↓, PPARα↑, CPT1α↑, UCP2↑	[74]
Baicalin	Scutellaria *baicalensis* Georgi (Huang qin)	HK2	Activation of CPT-1α target to attenuate inflammation	α-SMA↓, FN↓, TC↓, TG↓, LDL-C↓, HDL↑, IL-6↓, IL-1β↓, TNF-α↓, CPT1α↑	[75]
Berberine	*Rhizoma Coptidis* (Huang lian)	Podocytes	Activation of the AMPK and PGC-1α pathways to attenuate oxidative stress	CD36↓, CPT1↑, AMPK↑, PPARs↑, PGC-1α↑, FFA↓, TG↓, HDL↑	[76]
Morroniside	*Cornus officinalis* Siebold & Zucc. (Shan zhu yu)	TECs	Activation of the PGC-1α pathway to attenuate oxidative stress	PGC-1α↑, LXR↑, ABCA1↑, ABCG1↑, ApoE↑	[77]
4-O-Methylhonokiol	*Magnolia officinalis* (Hou pu)	GMCs	1. Activation of the AMPK/PGC-1α/CPT1B and Nrf2 pathways to attenuate oxidative stress 2. Inhibition of the TNF -κB pathway to attenuate inflammation	FN↓, LN↓, SOD2↓, CAT↓, AMPK↑, PGC-1α↑, CPT1B↑	[78]
Triptolide	*Tripterygium wilfordii* Hook.f. (Lei gong teng)	Podocytes	1. Inhibition of the NF-κB signaling pathway to attenuate inflammation 2. Inhibition of MCP-1 and 4-HNE expression to attenuate oxidative stress	MCP-1↓, 4-HNE↓, TC↓, TG↓, LDL↓, HDL↑, TNF-α↓, IL-1β↓, IL-6↓, IFN-γ↓	[79, 80]
Silymarin	*Silybum marianum*, *Asteraceae* family (Ru ji)	HK2	Inhibition of NOX4 and SOD2 expression to attenuate oxidative stress	NOX4↓, SOD2↓, CPT1α↓, CPT2↓, FFA↓, TC↓, TG↓, LDL↓	[81]
FYGL	*Ganoderma lucidum* (Ling zhi)	HBZY-1	Inhibition of the MAPK and NF-κB pathways to attenuate oxidative stress	NF-κB↓, TGF-β1↓, Col-IV↓, FN↓, TC↓, TG↓, LDL↓, HDL↑, NOX1↓, NOX4↓, SOD↑, CAT↑, GSH↑, MDA↓	[82]
Resveratrol	–	TECs	Inhibition of the JAML pathway and activation of the Sirt1 pathway to attenuate oxidative stress	JAML↓, SREBP-1↓, ChREBP↓, ADRP↓, Sirt1 ↑, TC↓, TG↓, LDL↓, HDL↑	[83]
18α-GA	Radix *Glycyrrhiza uralensis* Fisch*.* (Gan cao)	HK2, CMK-1	1. Activation of the Nrf2 pathway and inhibition of the MAPK pathway to attenuate oxidative stress 2. Inhibition of the NF-κB pathway to attenuate inflammation	HO-1↑, NQO-1↑, GCLM↑, SOD1↑, CAT↑, TNF-α↓, IL-6↓, IL-18↓, CCL-3↓, MCP1↓, PPARα↑, MCAD↑, CPT1α↑, UCP2↑, ChREBP↓, SREBF1↓, FASN↓, ACCα↓, SCD-1↓, FABP1↓, CD36↓	[84]

**Table 2:** Regulation of lipid metabolism by several Chinese medicines and their formulas

Drug	Components	Cell types	Mechanism	Effects	References
Astragali radix	*Astragalus membranaceus* (Fisch.) Bunge. (Huang qi)	MPC5	Activation of CPT1 targets to attenuate inflammation and oxidative stress	IL-2↓, IL-1β↓, MMP9↑, PPARα↑, CPT1α↑	[85]
Huangkui capsule	*Abelmoschus manihot* (L.) Medic. (Huang shu kui hua)	HepG2, HEK293T, HRMC	Inhibition of the IKKβ/NFκB signaling pathway to attenuate inflammation	aP2↑, LPL↑, CPT1↑, ACO↑, CYP4A↑, LDL↓, HDL↑, TGF-β ↓, Col-IV↓, TNF-α↓, IL-6↓, IL-1β↓, IL-2↓	[86]
Bupi Yishen Formula	*Astragalus membranaceus* (Fisch.) Bunge (Huang qi); *Codonopsis pilosula* (Franch.) Nannf. (Dang shen); *Atractylodes macrocephala* Koidz. (Bai zhu); *Poria cocos* (Schw.) Wolf (Fu ling); *Dioscorea opposita* Thunb. (Shan yao); *Coix lacryma-jobi* L. (Yi yi ren); *Cuscuta chinensis* Lam. (Tu si zi); *Salvia miltiorrhiza* Bunge (Dan shen)	HK2	Inhibition of the TGF-β1/Smad3 pathway to attenuate inflammation	FN1↓, Col1a1↓, Acta2↓, Vimentin↓, Col-1↓, α-SMA↓, E-cadherin↑, PPARα ↑, PGC-1α↑, CPT1α↑, CPT2↑, Acox1↑	[87]
Zishen Qingre Tongluo Formula	*Anemarrhena asphodeloides* Bunge (Zhi mu); *Gypsum Fibrosum* (Shi gao); *Ramulus Cinnamomi* (Gui zhi); Radix *Glycyrrhiza uralensis* Fisch*.* (Gan cao); C*oix lacryma-jobi* L. (Yi yi ren); *Dioscorea opposita* Thunb. (Shan yao)	TECs	Inhibition of the TGF-β1/Smad3 pathway to attenuate inflammation	TGF-β1↓, Smad3↓, Col- 1↓, FN↓, PGC-1α↑, PPARγ↑, PPARα↑	[88]
Fuxin Granules	*Anemarrhena asphodeloides* Bunge (Zhi mu); *Gypsum Fibrosum* (Shi gao); *Ramulus Cinnamomi* (Gui zhi); Radix *Glycyrrhiza uralensis* Fisch*.* (Gan cao); C*oix lacryma-jobi* L. (Yi yi ren); *Dioscorea Nipponica* Makino (Chuan shan long)	GMCs	Inhibition of the TGF-β1/Smad3, VEGFA, and VEGFR2 pathways to attenuate inflammation	TGF-β1↓, Smad2↓, Smad3↓, VEGFA↓, VEGFR2↓, eNOS↑, TC↓, TG↓, LDL↓, HDL↑	[89]
Zhenwu Decoction	*Aconitum carmichaeli var. carmichaeli* (Fu zi); *Poria cocos* (Schw.) Wolf (Fu ling); *Dioscorea opposita* Thunb. (Shan yao); *Zingiber officinale* Roscoe (Sheng jiang); *Atractylodes macrocephala* Koidz. (Bai zhu)	Renal fibroblasts	Inhibition of the TGF-β1 pathway and activation of the PPARγ pathway to attenuate oxidative stress	TGF-β1↓, PPARγ↑, LysoPC↓, LysoPE↓	[90]
Danggui Buxue Decoction	A*stragalus mongholicus* Bunge. (Huang qi); *Angelica sinensis* (Oliv.) Diels. (Dang gui)	GMCs, TECs	Attenuation of inflammation	TNF-α↓, IL-6↓, Degs2↓, Cers↓	[92]
Huangqi Decoction	A*stragalus mongholicus* Bunge. (Huang qi); *Poria cocos* (Schw.) Wolf (Fu ling); *Trichosanthes kirilowii* Maxim. (Gua lou); *Ophiopogon japonicus* (Thunb.) Ker Gawl. (Mai dong); *Schisandra chinensis* (Turcz.) Baill. (Wu wei zi); Radix *Glycyrrhiza uralensis* Fisch*.* (Gan cao); *Rehmannia glutinosa* (Gaertn.) DC. (Di huang)	Podocytes	Activation of the BMP pathway and inhibition of the Smad pathway to attenuate oxidative stress	BMP2↑, BMP7↑, BMPR-2↑, ERK↓, Smad1↓	[93]
Gandi Capsule	*Cornus officinalis* Siebold & Zucc. (Shan zhu yu); *Rehmannia glutinosa* (Gaertn.) DC. (Di huang); A*stragalus mongholicus* Bunge. (Huang qi); Leonurus japonicus Houtt. (Yi mu cao); *Scutellaria baicalensis* Georgi (Huang qin); *Styphnolobium japonicum* (L.) Schott (Huai hua); *Bombyx batryticatus* (Jiang can); *Phyllanthus Emblica* L. (Yu gan zi)	MPC5, Podocytes	Activation of the AMPK pathway to attenuate oxidative stress	Sirt1↑, HNF4A ↓, TG↓	[94]

#### Chinese medicine in regulating lipid metabolism

Various types of CM and their associated active ingredients can alleviate renal fibrosis by inhibiting the TGF-β/Smad signaling pathway. Dencichine, the main component of *Panax notoginseng* (San qi), corrects lipid metabolism disorders in diabetic nephropathy (DN) by reducing total cholesterol (TC), triglyceride (TG) and low-density lipoprotein (LDL) levels, as well as by increasing high-density lipoprotein (HDL) levels. Moreover, dencichine significantly inhibits the epithelial–mesenchymal transition (EMT), ECM accumulation, and glomerular mesangial matrix proliferation through inhibition of the TGF-β1/Smad signaling pathway [68]. Dioscin, the main component of *Dioscoreae Rhizoma* (Shan yao), regulates lipid metabolism by reducing the levels of TG and free fatty acids (FFAs) in renal tissue, downregulating the expression levels of sterol regulatory element-binding protein 1c (SREBP-1c), stearoyl-CoA desaturase-1 (SCD-1), and fatty acid synthase (FASN), and up-regulating the expression levels of acetyl-CoA carboxylase (ACC) and carnitine palmitoyltransferase 1 (CPT1). In addition, dioscin markedly upregulates sirtuin-3 (Sirt3) levels to regulate TGF-β1/Smad signaling, and it exerts an inhibitory effect on renal fibrosis [69]. Tartary buckwheat flavonoids (TBF) are active components extracted from Tartary buckwheat (*Fagopyrum tataricum* (L.) Gaertn.) (Ku qiao) that activate the AMPK/ACC pathway and inhibit the SREBP-1/FASN pathway to regulate blood lipid levels and reduce lipid deposition in the kidney. TBF also alleviate renal inflammation and renal fibrosis by inhibiting the TGF-β1/Smad signaling pathway and MAPK signaling pathway [70]. Total *Rhizoma Coptidis* alkaloids (TRCA) are extracted from *Rhizoma Coptidis* (Huang lian), and they can ameliorate lipid metabolism disorders by reducing TC and TG levels and increasing HDL levels in the serum. TRCA also ameliorate inflammation and oxidative stress in the kidney by inhibiting the advanced glycation end products receptor for advanced glycation end products (AGEs-RAGE)-TGF-β/Smad2 and PI3K-Akt pathways [71]. Ginsenoside Rg1 (GRg1) is one of the primary active ingredients of *Panax ginseng* C*.* A. Mey. (Ren shen). GRg1 reduces the excessive uptake of FFAs by glomerular mesangial cells (GMCs) and lipid deposition in the kidney by downregulating the expression of CD36 and posphorylation phospholipase C (p-PLC), and it also ameliorates glomerular fibrosis by inhibiting the activation of the TRPC6/NFAT2 and TGF-β/Smad2/3 pathways [72].

Rhein is an active anthraquinone isolated from *Rheum officinale* Baill. (Da huang); Juglanin is a natural compound extracted from the crude *Polygonum aviculare* L*.* (He shou wu), and baicalin is derived from the roots of Scutellaria *baicalensis* Georgi (Huang qin). The above three active ingredients used in CM upregulate the CPT1α-mediated oxidation of fatty acids to regulate lipid metabolism in the kidney. In particular, rhein inhibits renal tubular EMT, inflammation, and fibrosis by promoting CPT1α-mediated FAO through inhibition of the Sirt1/STAT3/Twist1 signaling pathway [73]. The protective effects of Juglanin against renal injury in human proximal tubular epithelial cells (HK2 cells) occur mainly through the suppression of the nuclear translocation of nuclear factor-kappa B (NF-κB) and histone deacetylase 3 (HDAC3); improving the expression of genes associated with FAO such as CPT1α, PPARα, and uncoupling protein 2 (UCP2) while markedly attenuating the mRNA levels of genes involved in fatty acid synthesis such as sterol regulatory element-binding factor 1 (SREBF1), FASN, SCD-1, ACCα and PPARγ; and repressing inflammation and lipid accumulation [74]. Baicalin ameliorates impaired FAO by targeting CPT1α and enhancing its expression, thereby attenuating renal fibrosis in DN. Both baicalin and juglanin reduce lipid droplet deposition and inflammatory responses in HK2 cells by inhibiting the expression of inflammatory factors such as TNF-α, IL-1β, and IL-6 [75].

Berberine, morroniside, and 4-O-methylhonokiol (MH) ameliorate renal lipid metabolism disorders by activating the PGC-1α pathway. Berberine is a plant alkaloid isolated from *Rhizoma Coptidis* (Huang lian) that promotes mitochondrial energy homeostasis and FAO in podocytes by activating the AMPK/PGC-1α pathway. In addition, berberine inhibits FFA uptake and lipid accumulation via downregulating the expression of CD36 and upregulating the expression of CPT1. Thus, oxidative stress and renal fibrosis are attenuated [76]. Morroniside, an iridoid glycoside isolated from *Cornus officinalis* Siebold & Zucc. (Shan zhu yu), reduces cholesterol accumulation in TECs by activating the PGC-1α pathway to upregulate the mRNA expression levels of its downstream target genes, namely, liver X receptor (LXR), ABCA1, ABCG1, and apolipoprotein E (ApoE) [77]. MH is a biologically active ingredient extracted from the stem bark of *Magnolia officinalis* (Hou pu) that ameliorates lipid metabolism by activating AMPK/PGC-1α/CPT1B-mediated FAO and Nrf2/SOD2-mediated anti-oxidative stress, thus exerting an anti-nephrogenic fibrotic effect [78].

Triptolide, an active diterpene purified from *Tripterygium wilfordii* Hook. (Lei gong teng), improves lipid metabolic disturbance by decreasing the levels of cholesterol, TG, and LDL, as well as by reducing lipid deposition in the kidneys. Triptolide also ameliorates oxidative stress and the inflammatory response in podocytes by decreasing the expression of MCP-1 and 4-HNE and inhibiting nuclear transcription factor NF-κB activation [79, 80]. Silymarin, which is derived from the plant milk thistle (*Silybum marianum*, in the *Asteraceae* family), significantly mitigates renal lipid accumulation and activates FAO in HK2 cells by downregulating CPT1α and CPT2 expression [81]. A proteoglycan isolated from *Ganoderma lucidum* (Ling zhi/FYGL) reduces the proliferation and accumulation of ECM in HBZY-1 cells induced by FFAs and regulates lipid metabolism by modulating blood lipid levels. Additionally, FYGL alleviates renal fibrosis by inhibiting the activation of the MAPK and NF-κB signaling pathways in the kidney [82].

Resveratrol (RSV) is a naturally occurring polyphenolic compound originating from different plants such as rhubarb, grapes, and peanuts. RSV downregulates the expression of SREBP-1, ChREBP, and ADRP through the JAML/Sirt1 lipid synthesis pathway, which in turn reduces renal lipid deposition and lipotoxic damage in the kidney [83]. The bioactive component 18-α-glycyrrhetinic acid (18α-GA) is extracted from Radix *Glycyrrhiza uralensis* Fisch*.* (Gan cao), and it reduces the mRNA expression levels of fatty acid synthesis and that of the uptake-related proteins ChREBP, SREBF1, FASN, CD36, SCD-1, and fatty acid binding protein 1 (FABP1) in HK2 cells. Additionally, 18α-GA markedly activates the phosphorylation level of ACCα (Ser79) and upregulates the expression of FAO-related molecules such as PPARα, MCAD, CPT1α, and UCP2, ultimately inhibiting lipid accumulation in HK2 cells [84].

*Astragali* radix (Huang qi/AR) reduces lipid peroxidation, ameliorates oxidative stress, and attenuates inflammatory responses in renal tissues by modulating glycerophospholipid, sphingolipid, and fatty acid metabolism and by acting on the key target CPT1 and the key metabolite carnitine [85]. Huangkui capsule (HKC), an extract from *Abelmoschus manihot* (L.) Medik., promotes FAO and reduces renal lipid accumulation by activating the transcriptional activity of PPARα and PPARγ and enhancing the expression levels of their target genes, including FABP4, lipoprotein lipase (LPL), acyl-CoA Oxidase (ACO), and cytochrome P450 4A (CYP4A). Moreover, HKC reduces endoplasmic reticulum stress and c-Jun NH2-terminal kinase activation in the kidney, which subsequently improves lipid metabolic disorders and renal injury [86].

Bupi Yishen formula (BYF) [87], Zishen Qingre Tongluo formula (ZQTF) [88], and Fuxin granules (FXG) [89] are three Chinese herbal compounds that correct lipid metabolism disorders in the kidney by inhibiting the TGF-β1/Smad3 pathway to attenuate the inflammatory response and inhibit renal fibrosis. FXG also attenuate kidney lipid droplet deposition by inhibiting the VEGFA/VEGFR2 pathway. Zhenwu decoction (ZWD) is a classic CM formula for the clinical treatment of renal diseases that regulates renal lipid metabolism, alleviates oxidative stress, and inhibits renal fibrosis by upregulating PPARγ signaling to reduce serum lysophospholipid levels [90]. The Shen-Shuai-II recipe (SSR) is an effective and safe formula clinically used for the treatment of CKD. SSR increases PPARα, CPT1α, CPT2, ACADL, and MCAD protein expression to improve lipid accumulation in NRK-52E cells. It also inhibits renal interstitial fibrosis by suppressing the inflammatory response of hypoxia-exposed TECs through PPARα-mediated FAO [91].

Danggui Buxue decoction (DBD), Huangqi decoction (HQD), and Gandi capsule (GC) are CM combinations commonly used to treat CKD, and these formulas also alleviate DN-induced abnormalities in renal lipid metabolism. DBD reduces the content of ceramide synthases (Cers), phosphatidylethanolamines, and phosphatidylcholines in the kidney by downregulating the transcription level of Degs2 and Cers, reducing lipid accumulation, and promoting Akt phosphorylation by upregulating the expression of alkaline ceramidases and pyruvate dehydrogenase kinase 1 [92]. HQD reduces the expression of CXCL16, a key mediator of lipid accumulation in glomeruli and tubules, and restores lipid metabolism balance by regulating bone morphogenetic protein (BMP) transcription and downstream targets, as well as by inhibiting extracellular signal-regulated kinase (ERK) phosphorylation, and Smad1 expression [93]. GC reduces TG and cholesterol levels in kidney and renal podocytes through the Sirt1/ AMPK/HNF4A pathway, thereby inhibiting lipid accumulation in the kidney [94].

**Fig. 6:**
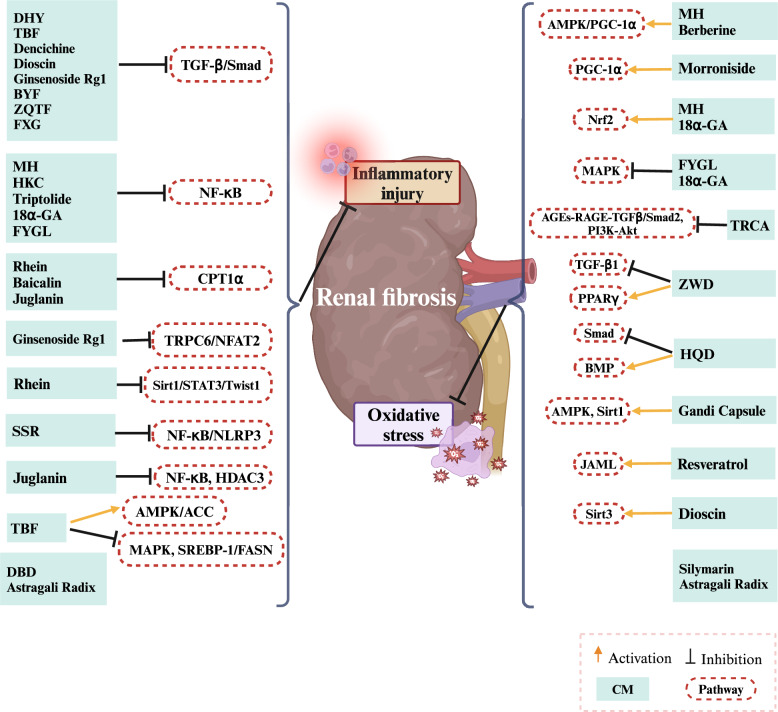
Signaling pathways involved in CMs and their active components that regulate lipid metabolism in renal fibrosis

Overall, we found that CM and its active components can promote FAO and reduce lipid deposition in renal tissues and cells, as well as reduce renal pathological damage, by activating or inhibiting various signaling pathways. This regulation of renal lipid metabolism helps to suppress renal inflammation and oxidative stress responses, thereby slowing the progression of CKD. Figure 6 shows the CMs and their active components that regulate lipid metabolism in the process of renal fibrosis, as well as the signaling pathways and mechanisms involved.

### Regulation of glucose metabolism

#### Activation of glycolysis promotes renal fibrosis

The kidney consumes a large amount of energy, and a normal and orderly energy metabolism system is the biochemical basis for maintaining the specific structure of this organ and its physiological functions. Proximal tubular cells account for 90% of the renal cortex and are the primary ATP-consuming cells in the kidney. Under normal physiological conditions, TECs are rich in mitochondria, and these cells mainly rely on FAO and mitochondrial OXPHOS to generate ATP. However, under hypoxic conditions, cells tend to generate energy through glycolysis rather than OXPHOS. This is especially true during renal fibrosis, in which cells require more energy to support their proliferation and the synthesis of large amounts of ECM. Hypoxia is a major factor triggering the progression of CKD and renal fibrosis [95, 96]. Because hypoxia stimulates glycolysis, glycolysis increases in response to the increased energy demand during renal fibrosis. Notably, increased glycolysis has been observed in renal metabolomics and RNA sequencing analyses of CKD patients [97].

When TECs are damaged, FAO activity is downregulated, and the major pathway of energy metabolism requires the upregulation of aerobic glycolysis to adapt to energy deficits; this metabolic reprogramming facilitates the maintenance of fibroblast proliferation and survival and promotes the development of renal fibrosis [98]. Aerobic glycolysis was first observed in tumor cells, and fibroblast activation and proliferation during renal fibrosis are similar to those observed in tumor cells [36]. TGF-β1 downregulates acetyl-CoA biosynthesis by inhibiting the activity of the pyruvate dehydrogenase complex, resulting in a decrease in protein acetylation that induces the metabolic shift of fibroblasts from OXPHOS to aerobic glycolysis and promotes renal fibroblast activation [99].

Reducing fibroblast activation by inhibiting glycolysis has become a commonly used approach to alleviate renal fibrosis [100]. For example, PKM2, a key enzyme in glycolysis associated with cell-dependent aerobic glycolysis, catalyzes the final step of glycolysis, which converts phosphoenolpyruvate to pyruvate [101]. A PKM2 agonist (TEPP-46) has been found to inhibit renal fibrosis by suppressing aberrant glycolysis and EMT activation [102]. Additionally, PKM2 activation reverses diabetes-induced glomerulopathy by increasing glucose metabolic flux, decreasing the levels of toxic glucose metabolites, and inducing mitochondrial biogenesis [103].

Therefore, understanding the relationship between renal fibrosis and glycolysis is essential for developing new therapeutic strategies. Recent studies have shown that alterations in cellular glucose metabolism play an important role in renal fibrosis, and some natural compounds from CM may represent new therapeutic avenues for renal fibrosis by targeting the glycolytic process.

#### Chinese medicine in inhibiting glycolysis

Shikonin is an aerobic glycolysis inhibitor isolated from *Arnebia euchroma* I. M. Johnst. (Zi cao). Shikonin inhibits renal aerobic glycolysis by reducing the phosphorylation of PKM2 and the production of FN and α-SMA in NRK-49F cells by inhibiting the TGF-β signaling pathway. Shikonin inhibits myofibroblasts activation, yielding anti-renal fibrosis effects [104]. *Sedum sarmentosum Bunge* (SSBE) (Chui pen cao), also known as sunflower, is an edible *Sedum* perennial herbaceous plant. The flavonoid substances in SSBE include quercetin, luteolin, kaempferol, isorhamnetin, and isoliquiritigenin. SSBE inhibits M1-macrophage polarization by reducing HIF-1α activity and inhibiting anaerobic glycolysis in macrophages, thus exerting a protective effect on M1-macrophage-mediated inflammatory injury in the kidney [105].

**Fig. 7:**
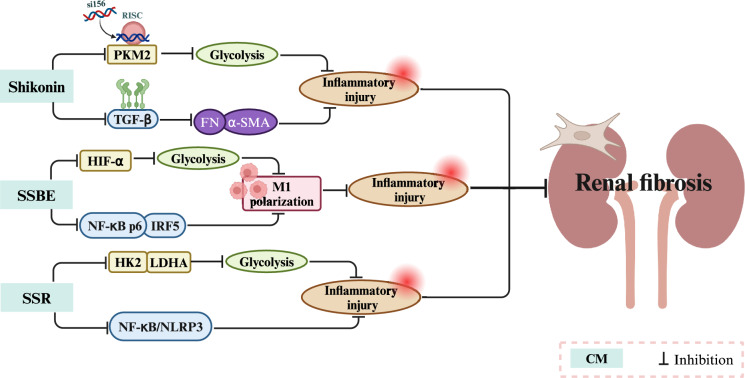
The mechanism by which CMs regulate glucose metabolism in renal fibrosis

SSR is a CM prescription that has demonstrated significant efficacy in treating CKD. In addition to regulating lipid metabolism to alleviate renal fibrosis, it also inhibits glycolysis by reducing the expression of HK2 and lactate dehydrogenase A (LDHA) in NRK-52E cells. This inhibition suppresses the activation of renal fibroblasts and the deposition of EMT, ultimately alleviating hypoxic renal injury and renal fibrosis [106]. Figure 7 and Table 3 show the regulatory effects of CM on glucose metabolism.

**Table 3:** Regulation of glucose metabolism and amino acid metabolism by several Chinese medicines and their active ingredients

Drug	Components	Cell Types	Mechanism	Effects	References
Shikonin	*Arnebia euchroma* I.M.Johnst. (Zi cao)	NRK-49F	1. Inhibition of glycolysis 2. Inhibition of the TGF-β pathway to attenuate inflammation	PKM2↓, FN↓, α-SMA↓	[104]
Sedum sarmentosum Bunge	*Sedum sarmentosum Bunge* (Chui pen cao)	NRK-52E, RAW264.7	1. Inhibition of glycolysis 2. Inhibition of the IRF5 and NF-κB p6 pathways to attenuate inflammation	MIF↓, MCP-1↓, IL-6↓, TNF-α↓, IL-12↓, iNOS↓	[105]
Shen Shuai II Recipe	*Codonopsis pilosula* (Franch.) Nannf. (Dang shen); *Sedum sarmentosum* Bunge (Yin yang huo); *Salvia miltiorrhiza* Bunge (Dan shen); *Angelica sinensis* (Oliv.) Diels. (Dang gui); *Rheum officinale* Baill. (Da huang); *Rhizoma Coptidis* (Huang lian); *Ligusticum wallichii* Franch. (Chuan xiong); *Prunus persica* (L.) Batsch (Tao ren); *Perilla frutescens* (L.) Britton (Zi su ye)	NRK-52 E	1. Inhibition of glycolysis 2. Promotion of FAO 3. Inhibition of the NF-κB/NLRP3 pathway to attenuate inflammation	IL-18↓, MCP-1↓, VEGF↓, PPARα↑, CPT1α↑, CPT2↑, ACADL↑, MCAD↑, HK2↓, LDHA↓, P-PKM↓, FN↓, α-SMA↓, Col-I↓, PCNA↓	[91, 106]
Total flavonoids in Epimedium	*Sedum sarmentosum* Bunge (Yin yang huo)	HK2	1. Regulation of glycine, serine, and threonine metabolism 2. Activation of the AMPK and Sirt 1 pathways and inhibition of the NF-κB pathway to attenuate inflammation and oxidative stress	TNF-α↓, IL-1β↓, SOD↑, GSH↑, CAT↑, MDA↓, α-SMA↓, FN↓, E-cadherin↑	[119]
SPX	Ootheca Mantidis (Sang piao xiao)	TECs	1. Inhibition of glutamine metabolism 2. Inhibition of the PTP pathway to attenuate inflammation and oxidative stress	Acta2↓, Col1a1↓, α-SMA↓, EGFR↓, NOD2↓, SOCS3↓	[120]
Danggui Shaoyao San	*Angelica sinensis* (Oliv.) Diels. (Dang gui); *Atractylodes macrocephala* Koidz. (Bai zhu); *Poria cocos* (Schw.) Wolf (Fu ling); Paeonia lactiflora Pall. (Shao yao); (Sam.) Juzep. (Zexie); *Ligusticum wallichii* Franch. (Chuan xiong)	GMCs, Podocytes	1. Regulation of glutamine, phenylalanine, alanine, and aspartate metabolism 2. Modulation of target TNF, IL-2, and ACE to attenuate inflammation	TNF-α↓, IL-2, ↓, ACE↓	[121, 122]

Renal fibrosis progression involves multiple cell types, signaling pathways, and metabolic alterations. Understanding the relationship between glycolysis and renal fibrosis is essential for developing new herbal therapeutic strategies. However, few studies have investigated the ability of CM to modulate glycolytic pathways and inhibit renal fibrosis. Further clinical and experimental research is needed to clarify the role of CM in regulating glycolysis in renal tissue.

### Regulation of amino acid metabolism

#### Amino acid metabolism and renal fibrosis

The kidney exerts significant effects on the regulation of AA levels in the body, including the synthesis, catabolism, filtration, reabsorption, and excretion of AAs [107]. The human kidney filters and reabsorbs approximately 70 g of AAs daily, which is particularly significant for the metabolism of AAs such as glutamine, proline, and tyrosine [108]. Because the kidneys are the primary organs involved in the excretion of glutamine and proline from arterial blood, they are also the main organs that release newly synthesized AAs, such as serine, tyrosine, and arginine. These AAs are produced in the kidney and subsequently exported to other tissues [109]. Specifically, the kidney is involved in the glucose synthesis of AAs, lactate, and pyruvate through the gluconeogenesis pathway [110]. Gluconeogenesis occurs primarily via glutamine, which contributes to 20–50% of glucose production to support its high energy requirements [111–113]. The kidney regulates nitrogen and acid–base balance in the body by breaking down glutamine. When renal fibrosis occurs, the resulting impairment of renal function may lead to azotemia and acidosis [108]. Glutamine is one of the most abundant free AAs in the human body, and it can replace glucose in the TCA cycle under hypoxic conditions. It also exerts an important effect on various physiological processes, including the synthesis of DNA, RNA, proteins, aminosaccharides, and glucose [114].

AAs play an irreplaceable intermediary role between glucose metabolism and lipid metabolism in cells, and this intermediary role is particularly essential in renal diseases, especially in renal fibrosis in CKD. When kidney injury occurs, some AAs, such as glutamine, glycine, and arginine, may become overaccumulated or deficient. This AA metabolic imbalance affects cell signal transduction, resulting in oxidative stress and inflammatory responses, thus exacerbating renal fibrosis progression [115, 116]. Therefore, the targeted regulation of AA metabolism is another promising approach for effectively inhibiting renal fibrosis. For example, silencing GLS can inhibit glutamine catabolism, which in turn inhibits fibroblast activation and ECM accumulation via the mTOR/MTFP1/DRP1 pathway, thereby slowing renal fibrosis progression [45]. In addition, increasing the expression of glycine N-methyltransferase (GNMT) can reduce the phosphorylation levels of TNF-α, IL-1β, and P65 and downregulate the expression of Col-I, α-SMA, and FN, thereby alleviating inflammation and fibrosis in DN [117]. Furthermore, activating the arginine metabolic pathway can increase spermidine levels in the kidney and activate the nuclear factor erythroid 2-related factor 2 (Nrf 2) pathway, thereby reducing inflammation, oxidative stress, and fibrosis in TECs [118].

#### Chinese medicine in regulating amino acid metabolism

Recent studies have revealed that some CMs and their effective compounds can reprogram AA metabolism and inhibit renal fibrosis. The total flavonoids in epimedium (TFE) are the main bioactive components of *Sedum sarmentosum* Bunge (Yin yang huo). TFE regulate the glycine, serine, and threonine metabolic pathways to promote AMPK activation and regulate the AMPK/ACC and AMPK/Sirt1/NF-κB pathways. In turn, TFE improve renal inflammation and oxidative stress and inhibit HK2 cells from undergoing EMT, thereby reducing renal fibrosis [119]. Ootheca Mantidis (SPX) is a commonly prescribed CM for CKD that meliorates apoptosis and inflammation by suppressing glutamine catabolism and downregulating the expression of EGFR, NOD2, and SOCS3 in the peptidyl-tyrosine phosphorylation (PTP) pathway. Additionally, SPX inhibits the expression of fibrosis-related genes, collectively contributing to the alleviation of renal fibrosis [120].

**Fig. 8:**
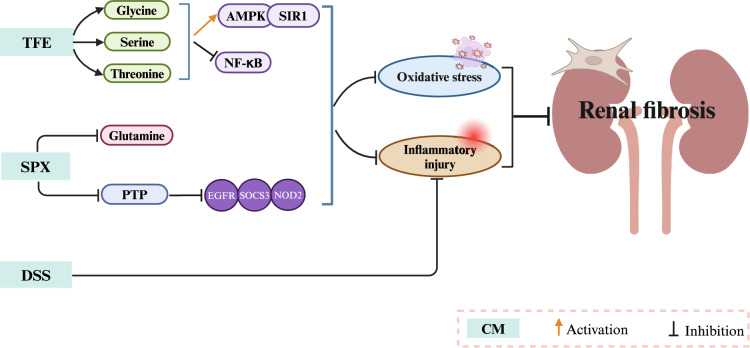
The mechanism by which CMs regulate amino acid metabolism in renal fibrosis

Danggui Shaoyao San (DSS) is a prepared compound that effectively reduces renal fibrosis [121]. DSS mainly regulates TNF-α, IL-2, and angiotensin-converting enzyme (ACE) to interfere with metabolites such as L-glutamine and L-phenylalanine, subsequently modulating the metabolic pathways of glutamine, phenylalanine, alanine, and aspartic acid to alleviate renal inflammation and exert renoprotective effects [122]. Figure 8 and Table 3 show the regulatory effects of CM on AA metabolism.

In summary, targeted regulation of AA metabolism by CM is an effective approach for inhibiting renal fibrosis. However, it is important to note that the impact of AA metabolism on renal fibrosis involves various biological processes and pathways, leading to different effects under different conditions. Renal fibrosis, a pathological feature of CKD, involves the excessive accumulation of ECM, which can cause sustained damage to the renal structure and to renal function. AA metabolism plays a bidirectional role in this process, with its regulation depending on several factors, including the type and quantity of AA intake, the metabolic state of the kidneys, and the progression of the disease. Our understanding of the relationship between AA metabolism and renal fibrosis remains incomplete, necessitating further basic and clinical studies to elucidate this complex interaction.

## Conclusion

**Fig. 9:**
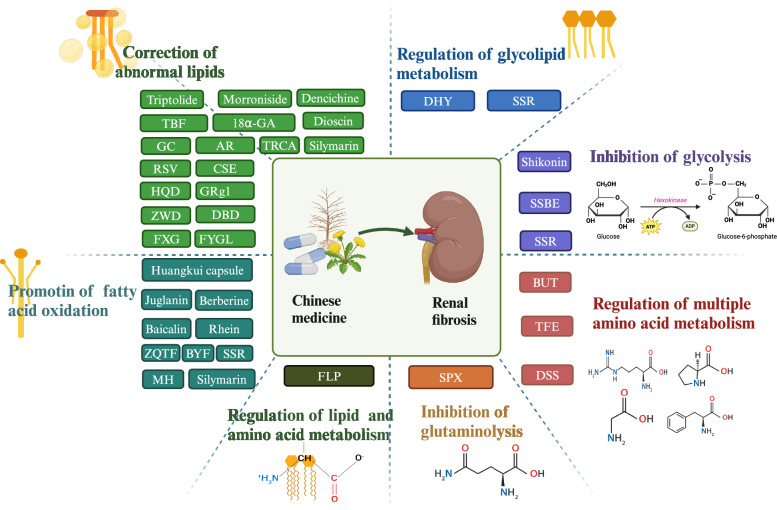
CMs and their active ingredients that inhibit renal fibrosis through metabolic reprogramming

This review, which focuses on metabolic reprogramming, is the first to systematically integrate lipid metabolism, glycolysis, and amino acid metabolism to provide a comprehensive exploration of their roles and interrelationships in renal fibrosis. This multidimensional perspective on metabolic regulation offers a novel theoretical framework with the aim of better understanding the pathological mechanisms underlying renal fibrosis. Furthermore, this paper is the first to reveal the specific regulatory mechanisms of CM and its active components across multiple metabolic pathways. Notably, by targeting lipid metabolism, glycolysis, and amino acid metabolism, CM modulates signaling pathways such as those associated with TGF-β/Smad, MAPK, PGC-1α, STAT3, NF-κB, Nrf-2, and AMPK. This modulation impacts targets such as TNF-α, IL-1β, IL-6, CD36, SREBP, CPT-1, ChREBP, SOD1, and CAT, and it also mitigates mechanisms such as oxidative stress and inflammation. Ultimately, these actions exert an inhibitory effect on renal fibrosis, further confirming the synergistic effects of CM through its multi-target, multi-mechanism approach. The metabolic reprogramming pathways revealed in this study, and particularly the regulation of glycolysis and amino acid metabolism, provide novel therapeutic targets for the treatment of renal fibrosis. These findings not only complement existing therapeutic approaches, but they may also pave the way for new treatment avenues, optimizing comprehensive strategies for managing renal fibrosis. Figure 9 summarizes the CMs and their active ingredients that inhibit renal fibrosis through metabolic reprogramming.

## Prospects

Studies on regulating cellular metabolic reprogramming through CM to inhibit renal fibrosis have primarily focused on lipid metabolism. However, few studies have investigated the potential of CM to inhibit renal fibrosis by targeting glucose and AA metabolism, which are crucial components of metabolic reprogramming. Conducting in-depth clinical and experimental research on the regulation of glucose and amino acid metabolism by CM to modulate renal fibrosis will enhance our comprehensive understanding of CM’s role in metabolic reprogramming and its effect on renal fibrosis.

Future research should further focus on the metabolic reprogramming of macrophages and T cells in the process of renal fibrosis, exploring their regulatory effects on immune and inflammatory processes within the renal fibrosis microenvironment. In addition to classical metabolic pathways, the roles of lactate metabolism, the polyol pathway, the pentose phosphate pathway, and one-carbon metabolism in renal fibrosis remain unclear and warrant further investigation. Moreover, the interplay between metabolic reprogramming and epigenetic modifications presents an intriguing area of research. Nearly all epigenetic modifications—such as acetylation, methylation, succinylation, β-hydroxybutyrylation, and lactylation—depend on metabolites. Conversely, epigenetic modifications can regulate the enzymes and proteins involved in metabolism. What roles do these modifications play in the development of renal fibrosis? Can CM offer effective interventions in this context? Both of these questions are challenging, and addressing them will be highly valuable for future research; they thus represent important directions for future clinical and experimental studies. We believe that exploring these issues in depth will further expand the potential applications of CM in the treatment of renal fibrosis.

## Supplementary Information

Additional file 1.

## Data Availability

Not applicable.

## Abbreviations

AA: Amino acid
OXPHOS: Oxidative phosphorylation
HIF-1α: Hypoxia-inducible factor -1α
ECM: Extracellular matrix
PFKFB3: 6-Phosphofructo-2-kinase/fructose-2,6-biphosphatase 3
TGF-β: Transforming growth factor β
P38-MAPK: P38 mitogen-activated protein kinase
FAO: Fatty acid oxidation
PGC-1α: Peroxisome proliferator-activated receptor γ coactivator 1α
HK3: Hexokinase 3
FSTL1: Follistatin-like protein 1
PKM2: The pyruvate kinase M2
AMPK: AMP-activated protein kinase
PI3K/Akt: Phosphatidylinositol 3-kinase /protein kinase B
CKD: Chronic kidney disease
TEC: Tubular epithelial cell
ATP: Adenosine triphosphate
HK2: Hexokinase 2
TCA: Tricarboxylic acid cycle
GLS: Glutamine synthetase
FN: Fibronectin
α-SMA: Alpha-smooth muscle actin
BCAA: Branched-chain amino acid
PPARγ: Peroxisome proliferator-activated receptor gamma
CM: Chinese medicine
eNOS: Endothelial-type nitric oxide synthase
PPARα: Peroxisome proliferator-activated receptor alpha
IL: Interleukin
FLP: The surface layer of Poria Cocos
BUT: N-butanol extract
DHY: Rehmannia glutinosa leaves total glycoside
DN: Diabetic nephropathy
TC: Total cholesterol
TG: Triglyceride
LDL: Low-density lipoprotein
HDL: High-density lipoprotein
EMT: Epithelial-mesenchymal transition
FFA: Free fatty acid
SREBP: Sterol regulatory element-binding protein
SCD-1: Stearoyl-CoA desaturase 1
FASN: Fatty acid synthase
ACC: Acetyl-CoA carboxylase
CPT1: Carnitine palmitoyltransferase 1
Sirt3: Sirtuin 3
TBF: Tartary Buckwheat Flavonoids
TRCA: Total Rhizoma Coptidis Alkaloids
GRg1: Ginsenoside Rg1
GMC: Glomerular mesangial cell
p-PLC: Phosphorylation phospholipase C
NF-κB: Nuclear factor-kappa B
HK2 cell: Human proximal tubular epithelial cell
HDAC3: Histone deacetylase 3
UCP2: Uncoupling protein 2
SREBF1: Sterol regulatory element-binding factor 1
MH: 4-O-methylhonokiol
LXR: Liver X receptors
ApoE: Apolipoprotein E
FYGL: A proteoglycan extracted from Ganoderma Lucidum
RSV: Resveratrol
18α-GA: 18α-Glycyrrhetinic acid
FABP: Fatty acid binding protein
HKC: Huangkui Capsule
LPL: Lipoprotein lipase
ACO: Acyl-CoA Oxidase
CYP4A: Cytochrome P450 4A
BYF: Bupi Yishen Formula
ZQTF: Zishen Qingre Tongluo Formula
FXG: Fuxin Granules
ZWD: Zhenwu Decoction
SSR: Shen Shuai II Recipe
DBD: Danggui Buxue Decoction
HQD: Huangqi Decoction
GC: Gandi Capsule
Cers: Ceramide synthases
BMP: Bone morphogenetic protein
ERK: Extracellular regulated protein kinase
SSBE: Sedum Sarmentosum Bunge
LDHA: Lactate dehydrogenase A
TFE: Total flavonoids in Epimedium
SPX: Ootheca Mantidis
PTP: Peptidyl-tyrosine phosphorylation pathway
DSS: Danggui Shaoyao San
ACE: Angiotensin-converting enzyme
