# Supplementary material for: Metabolic reprogramming and renal fibrosis: what role might Chinese medicine play?
Source: Chin Med. 2024 Oct 28;19:148. doi: 10.1186/s13020-024-01004-x (PMC11514863; doi:10.1186/s13020-024-01004-x)
Supplement: Supplementary file 1 — Additional file 1. [file 13020_2024_1004_MOESM1_ESM.pdf]

CERTIFICATE

OF ENGLISH LANGUAGE EDITING

Metabolic Reprogramming and Renal Fibrosis: What Role Might Chinese Medicine Play?

Metabolic reprogramming is a pivotal biological process in which cellular metabolic patterns change to meet the energy demands of increased cell growth and proliferation. In this review, we explore metabolic reprogramming and its impact on fibrotic diseases, providing a detailed overview of the key processes involved in the metabolic reprogramming of renal fibrosis, including fatty acid decomposition and synthesis, glycolysis, and amino acid catabolism. In addition, we report that Chinese medicine ameliorates renal inflammation, oxidative stress, and apoptosis in chronic kidney disease by regulating metabolic processes, thereby inhibiting renal fibrosis. Furthermore, we reveal that multiple targets and signaling pathways contribute to the metabolic regulatory effects of Chinese medicine. In summary, this review aims to elucidate the mechanisms by which Chinese medicine inhibits renal fibrosis through the remodeling of ...

This document certifies that the manuscript listed above was copy edited for English language by LetPub, with regard to grammar, punctuation, spelling, and clarity. Documents receiving this certification should be regarded as having undergone professional editorial revision for English language before submission. However, the authors may accept or reject LetPub's suggestions and changes at their own discretion and LetPub does not have editorial control over the submitted documents. Submitted documents may have new text that was not provided to LetPub for review. Please use the verification link below to determine the validity of the submitted version.

September 10, 2024

Date of Revision

LetPub

LetPub is an author service brand owned and operated by Accdon LLC.
Tel: 1-781-202-9968 Email: info@accdon.com
Address: 400 Fifth Ave, Suite 530, Waltham, MA 02451, United States

This manuscript has been individually edited for grammar, punctuation, spelling, and clarity. You may verify the authenticity of this certificate on our website (https://www.letpub.com/editorial-certificate) at any time using this manuscript's unique code: PR_240418U338F v240905.

© 2024 Accdon, LLC.
